# The relationship between childhood adversity and sleep quality among rural older adults in China: the mediating role of anxiety and negative coping

**DOI:** 10.1186/s12888-024-05792-2

**Published:** 2024-05-08

**Authors:** Yuqin Zhang, Chengwei Lin, Hongwei Li, Lei Li, Xueyan Zhou, Ying Xiong, Jin Yan, Mengxue Xie, Xueli Zhang, Chengchao Zhou, Lian Yang

**Affiliations:** 1 School of Public Health, Chengdu University of Traditional Chinese Medicine, Chengdu, 610075 Sichuan China; 2 Sichuan Provincial Center for Disease Control and Prevention, No.6, Zhongxue Road, Wuhou District, Chengdu, 610041 China; 3 Hospital of Chengdu University of Traditional Chinese Medicine, Deyang Integrated Traditional Chinese and Western Medicine Hospital, Deyang, 618000 China; 4 Centre for Aging Health Service of Deyang City, Deyang, 618000 China; 5 Health Commission of Deyang City, Deyang, 618000 China; 6 Sichuan Provincial Health Information Center, Chengdu, 610015 Sichuan China; 7 Centre for Health Management and Policy Research,School of Public Health, Cheeloo College of Medicine,Shandong University, NHC Key Lab of Health Economics and Policy Research, Shandong University, Jinan, 250012 China

**Keywords:** Rural older adults, Sleep quality, Childhood adversity experiences, Anxiety, Negative coping, Chain mediation

## Abstract

**Background:**

Studies have revealed the effects of childhood adversity, anxiety, and negative coping on sleep quality in older adults, but few studies have focused on the association between childhood adversity and sleep quality in rural older adults and the potential mechanisms of this influence. In this study, we aim to evaluate sleep quality in rural older adults, analyze the impact of adverse early experiences on their sleep quality, and explore whether anxiety and negative coping mediate this relationship.

**Methods:**

Data were derived from a large cross-sectional study conducted in Deyang City, China, which recruited 6,318 people aged 65 years and older. After excluding non-agricultural household registration and lack of key information, a total of 3,873 rural older adults were included in the analysis. Structural equation modelling (SEM) was used to analyze the relationship between childhood adversity and sleep quality, and the mediating role of anxiety and negative coping.

**Results:**

Approximately 48.15% of rural older adults had poor sleep quality, and older adults who were women, less educated, widowed, or living alone or had chronic illnesses had poorer sleep quality. Through structural equation model fitting, the total effect value of childhood adversity on sleep quality was 0.208 (95% CI: 0.146, 0.270), with a direct effect value of 0.066 (95% CI: 0.006, 0.130), accounting for 31.73% of the total effect; the total indirect effect value was 0.142 (95% CI: 0.119, 0.170), accounting for 68.27% of the total effect. The mediating effects of childhood adversity on sleep quality through anxiety and negative coping were significant, with effect values of 0.096 (95% CI: 0.078, 0.119) and 0.024 (95% CI: 0.014, 0.037), respectively. The chain mediating effect of anxiety and negative coping between childhood adversity and sleep quality was also significant, with an effect value of 0.022 (95% CI: 0.017, 0.028).

**Conclusions:**

Anxiety and negative coping were important mediating factors for rural older adult’s childhood adversity and sleep quality. This suggests that managing anxiety and negative coping in older adults may mitigate the negative effects of childhood adversity on sleep quality.

## Background

The global population is entering an aging stage, and China has the fastest rate of population aging in the world. According to China’s seventh national census, in 2020, 191 million individuals were aged 65 years and older, accounting for 13.50% of the total population [1], and the proportion of people aged 65 and above in rural is 6.6% higher than in urban [2]. In addition, China’s long-standing urban-rural dual structure has resulted in inequality in economic, medical, and educational development, leading to significant differences in the health status of China’s urban and rural older populations [3, 4]. Relevant studies have found that, urban residents have a higher survival rate [5], better self-assessed health status and better self-assessed self-care ability than rural dwellers [6]. Therefore, to reduce health inequalities among older adults, the health status of rural older adults is an important focus.

Good quality sleep has been found to be essential for health [7–9]. However, sleep problems are prevalent among the older population [10, 11]. Gulia and Tatineny have reported that the current prevalence of sleep disorders in the global older population is 30–40% [12, 13]. In a systematic review, Lu reported that the overall prevalence of poor sleep among the older population in China had reached 35.9% [14]. In the rural older adults, the prevalence of sleep disorders is more than 40% [15], even as high as 58.40% [16].There are various factors that affect sleep quality [17, 18]. Adverse childhood experiences (ACEs) are stressful and/or traumatic experiences that occur during childhood [19]. There is growing evidence that ACEs may lead to sleep problems in adulthood [20, 21] and that the influence can last up to 50 years [22]. For example, emotional abuse and neglect experienced early in life impede the development of individuals’ social relationships later in life and negatively affect the subjective sleep quality of older adults [23]. A study by Dorji. found that older adults with multiple (≥ 7) ACEs had a higher incidence of insomnia [24]. Although previous investigations have indicated the relationship between childhood adversity and sleep quality in older adults, they have ignored possible potential mechanisms for this relationship.

Previous studies have found that anxiety negatively affects sleep quality in older adults [25], whereas a good mental state can improve their sleep quality. Notably, childhood adversity may be associated with increased anxiety symptoms in late adulthood [26]. Raposo have reported that older adults who experienced childhood adversity were more likely to suffer from anxiety (OR = 1.48; 95%CI = 1.20–1.83) [27]. Considering the relationships among anxiety, childhood adversity, and sleep quality, one aim of this study was to verify whether anxiety mediates the relationship between childhood adversity and sleep quality.

A coping style refers to a psychological and behavioral strategy adopted by an individual in response to changes in the internal and external environment [28]. Negative coping is usually positively associated with sleep disorders [29, 30]. Coping style usually evolves over time and may be influenced by exposure to childhood adversity; for instance, people exposed to early adverse experiences show predominantly emotion-focused and avoidance coping styles, such as denial and disengagement [31, 32]. In addition to childhood adversity, negative emotions or psychological states also can influence individuals’ coping strategies [33]. For example, Orgeta reported that older adults with high levels of anxiety were more likely to adopt dysfunctional coping [34]. Therefore, we hypothesized that anxiety affects coping styles in older adults and that negative coping may be a potential mediator between childhood adversity and sleep quality.

Stress is defined as the process of adaptive and coping responses when an individual faces or perceives threatening or challenging environmental changes [35]. People respond to stress with either problem-focused coping or emotion-focused coping [36]. Stress can be caused by many factors, such as early adversities, and the result of stress is adaptive or maladaptive psychosomatic responses. Based on the above, we constructed a structural equation model of a large cross-sectional dataset to explore the effects of childhood adversity on sleep quality, with childhood adversity as the stressor and anxiety and negative coping as mediators.

## Research methods

### Research population

The data were sourced from a large-scale cross-sectional study conducted in 2022 that recruited older adults aged 65 years and older living in 6 districts and counties in Deyang City, Sichuan Province. Using a multistage stratified random cluster sampling method, townships (streets) were randomly selected from six county (districts), administrative villages (communities) were randomly selected from each sample township (streets), finally, people over 65 years old were selected randomly in each chosen village or community. The inclusion criteria were as follows: (1) individuals aged ≥ 65 years; (2) permanent residents in the survey area (those who have lived in the area for 6 months or more); (3) those who signed an informed consent form and agreed to take the questionnaire survey. The exclusion criteria were as follows: (1) unwilling to participate in research; (2) individuals identified by local village doctors who are unable to answer questions independently and have a history of dementia;3) other reasons for not participating in the study. The household registration system is a very important factor affecting the unequal social welfare rights and privileges of urban and rural residents in China [37], which is associated with poor health [38]. In this study, rural means that residents with agricultural household registration. A total of 6318 respondents were recruited, excluding non-agricultural household registration (2345) and missing main information (100), and finally included 3873 for analysis. The study was approved by the Medical Ethics Committee of the Affiliated Hospital of Chengdu University of Chinese Medicine, and all participants signed an informed consent form before taking the survey.

### Measurement tools

#### General information

This includes the age, gender, education level, marital status, chronic disease status, and exercise status of the participating older adults.

#### Childhood adversity

Childhood adversity was measured using the Adverse Childhood Experiences Scale developed by the Centers for Disease Control and Prevention (USA). The scale contains three major dimensions (abuse, neglect, and household dysfunction) and ten subdimensions including emotional abuse, physical abuse, sexual abuse, and emotional neglect. Higher ACE scores indicate more severe ACE exposure [19, 39]. The internal consistency coefficients of the abuse, neglect, and household dysfunction subscales in this study were 0.790, 0.732, and 0.778, respectively.

#### Sleep quality

Sleep quality was evaluated using the revised Chinese-version Pittsburgh Sleep Quality Index (PSQI). The scale consists of seven dimensions including subjective sleep quality, sleep latency, sleep duration, sleep efficiency, sleep disturbance, use of sleep medication, and daytime dysfunction. A PSQI score of ≥ 7 is generally considered to indicate poor sleep quality [15, 40, 41]. The internal consistency coefficient of the scale in this study was 0.754.

#### Anxiety

Anxiety in older adults was measured using the Self-Rating Anxiety Scale (SAS). The scale consists of 20 items and is rated on a 4-point scale. An SAS score of 50 or more is considered to be indicative of anxiety symptoms [42]. The internal consistency coefficient of this scale in this study was 0.831.

#### Trait coping style

Negative coping was measured using the Trait Coping Style Questionnaire (TCSQ). The scale consists of 20 questions in 2 dimensions—negative coping and positive coping—and is rated on a 5-point scale. The negative coping and positive coping scores are the sum of the scores for each item in the corresponding dimensions. A positive total score indicates a predominantly positive attitude toward coping with events, whereas a negative score indicates a predominantly negative coping style [43, 44]. Only the negative coping dimension of the scale, which has an internal consistency coefficient of 0.929, was selected in this study.

### Statistical analysis

The variables in the study were descriptively analyzed using the mean, standard deviation, frequency (n), and constituent ratio (%), and difference tests were conducted using t-tests and the Kruskal-Wallis H test. Spearman’s correlation was used to analyze whether there were correlations between sleep quality and the variables. Finally, a multiple-mediator structural equation model was constructed to analyze the effects of anxiety and negative coping on the relationship between sleep quality and childhood adversity, and the bootstrap method was applied to verify the mediating effect. After the initial establishment of the model, we evaluated the fit degree of the structural equation model and adjusted the model via calculating indicators such as standardized root-mean-square residual (SRMR ≤ 0.08), root-mean-square error of approximation (RMSEA ≤ 0.08), goodness of fit index (GFI ≥ 0.90), comparative fit index (CFI ≥ 0.90), normed fit index (NFI ≥ 0.90) according to the studies by Wen and Kang [45, 46]. Data were analyzed using SPSS 25.0 and AMOS 24.0 software, and a P value < 0.05 was considered to be statistically significant. The bootstrap CI was set to 95%, and the bootstrap sample size was 5000. If the 95% CI interval does not contain 0, it indicates a significant mediating effect.

### Research results

#### Comparison of the general information and sleep quality scores of the study participants

A total of 3,873 older adults were included in this study. The mean participant age was 72.84 ± 6.13 years, ranging from a minimum of 65 years to a maximum of 99 years. The mean PISQ score was 6.94 ± 3.88, and older adults with poor sleep quality (PSQI score ≥ 7) accounted for 48.15%. The mean ACE score was 2.09 ± 1.16, the mean SAS score was 44.13 ± 9.84, and the mean TCSQ negative coping score was 21.88 ± 8.23.

The results of univariate analysis showed that among the different gender populations, women had poorer sleep quality and a statistically significantly higher PSQI score than men at 7.44 ± 3.98 (t = 8.845, *p* < 0.001). The PSQI score increased with age: that of adults aged 80 years and older was 7.32 ± 4.01, and the difference was statistically significant (H = 11.125, *p* = 0.004). Regarding the groups with different educational levels, the highest PSQI score was found among illiteracy individuals (7.39 ± 4.01), with a statistically significant difference (H = 39.885, *p* < 0.001). Sleep quality varied among older adults with different marital statuses, and the worst sleep quality was found in widowed older adults, with a PSQI score of 7.52 ± 4.00, which presented a statistically significant difference (H = 39.582, *p* < 0.001). Older adults living alone had the worst sleep quality with a statistically significantly different PSQI score of 7.46 ± 3.90 (H = 20.904, *p* < 0.001). Older adults with chronic diseases had poor sleep quality with a statistically significantly different PSQI score of 7.4 ± 3.95 (t=-8.83, *p* < 0.001) (Table 1).

**Table 1:** Comparison of sleep quality among the elderly by different sociodemographic characteristics in Deyang City, Sichuan Province, China in 2022

Characteristics	*N*(%)	PSQI score ≥ 7 *n*(%)	PSQI score (x ± s)	t/H	*P*	Paired comparison
Total	3873(100)	1865(48.15)	6.94 ± 3.88	——	——	——
Gender						
Female	2080(53.7)	1118(59.9)	7.44 ± 3.98	8.845	<0.001	
Male	1793(46.30)	747(40.1)	6.36 ± 3.68			
Age						
65~	1434(37.03)	655(35.12)	6.69 ± 3.82	11.125	0.004	①<③
70~	1846(47.66)	910(48.79)	7.01 ± 3.88			
≥ 80	593(15.31)	300(16.09)	7.32 ± 4.01			
Degree of education						
Illiteracy	1696(43.80)	899(48.20)	7.39 ± 4.01	39.885	<0.001	②<①, ③<①
Primary school	1715(44.30)	777(41.66)	6.64 ± 3.74			
Middle school	393(10.10)	161(8.63)	6.40 ± 3.70			
University and above	69(1.80)	28(1.50)	6.25 ± 4.06			
Marital status						
Not married	55(1.40)	19(1.02)	5.69 ± 3.58	39.582	<0.001	①<③, ②<③
Married	2695(69.60)	1231(66.01)	6.73 ± 3.81			
Widowed	1101(28.40)	605(32.44)	7.52 ± 4.00			
Divorce	22(0.60)	10(0.54)	6.41 ± 4.28			
Living condition						
Alone	623(16.10)	346(18.55)	7.46 ± 3.90	20.904	<0.001	②<①, ③<①
With spouse	1543(39.80)	697(37.37)	6.75 ± 3.86			
With children	819(21.10)	389(20.86)	6.71 ± 3.81			
With spouse and children	888(22.90)	433(23.22)	7.12 ± 3.93			
Chronic disease						
No	1626(42.00)	677(36.30)	6.31 ± 3.68	-8.83	<0.001	
Yes	2247(58.00)	1188(63.70)	7.40 ± 3.95			

### Association of sleep quality with childhood adversity, anxiety, and negative coping in rural older adults

The relevant analysis results indicated that the PSQI score was positively correlated with the ACE score (*r* = 0.092, *P* < 0.01). The PSQI score was positively correlated with the SAS score and negative coping score (*r* = 0.279 and *r* = 0.239, respectively; both *P* < 0.01). The ACE score was positively correlated with the SAS score and negative coping score (*r* = 0.217 and *r* = 0.133, respectively; both *P* < 0.01). There was also a positive correlation between the SAS score and negative coping score (*r* = 0.351, *P* < 0.01) (Table 2).

**Table 2:** Results of Spearman correlation analysis among variables

Variables	PSQI	ACEs	SAS	Negative coping styles
PSQI	1			
ACEs	0.092**	1		
SAS	0.279**	0.217**	1	
Negative coping styles	0.239**	0.133**	0.351**	1

### Analysis of mediating effects

#### Goodness-of-fit indices and path coefficients for the theoretical model of older adults’ sleep quality

**Fig. 1:**
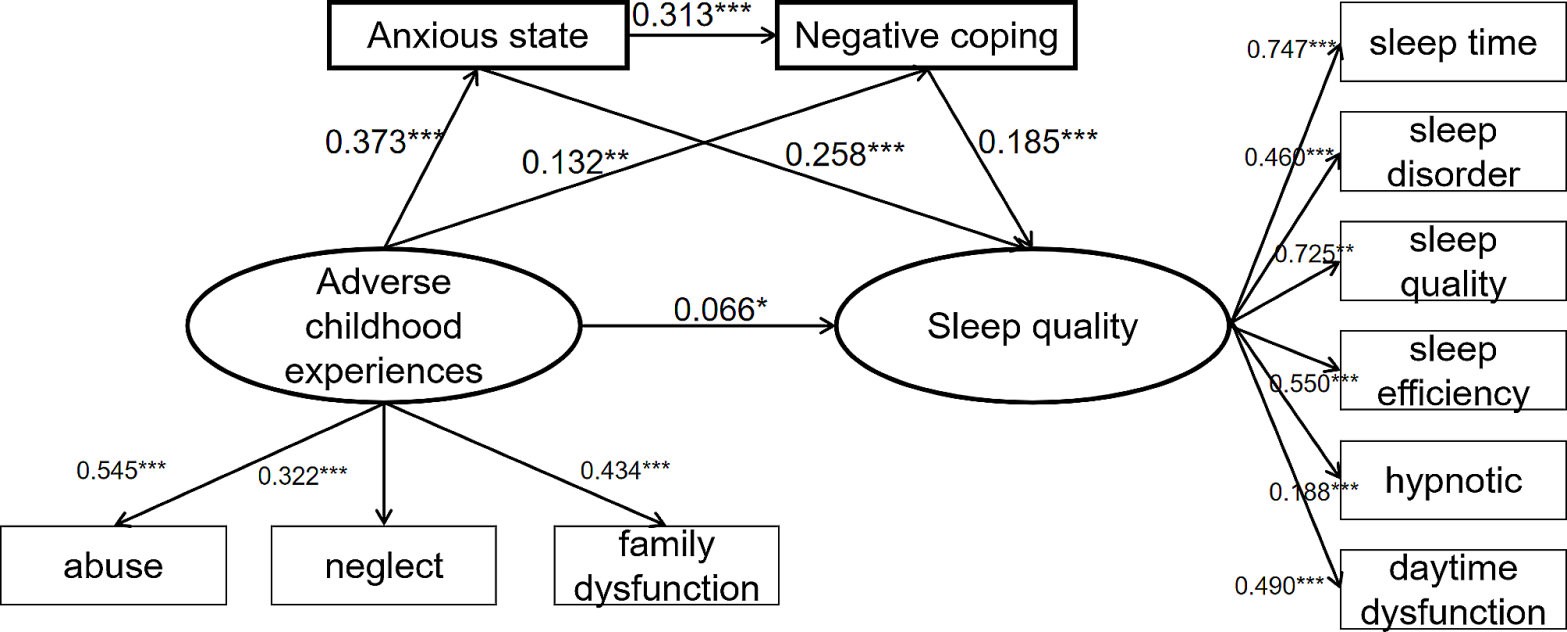
Serial mediation models for childhood adversity, anxiety, negative coping and sleep quality

Based on the results of the above analyses, a structural equation model was constructed with childhood adversity as the independent variable, anxiety and negative coping as the mediating variables, and sleep quality as the dependent variable. The final model was screened according to the following model fitting indices: SRMR = 0.05, RMSEA = 0.06, GFI = 0.97, CFI = 0.90 and NFI = 0.89. The results of the fitting indices indicated that the model was well fitted. The differences in each of the standardized path coefficients in the model were statistically significant (all *P* < 0.05) (Fig. 1).

### Bootstrap test of the theoretical model of older adults’ sleep quality

Table 3 demonstrates the results of structural modeling: (1) The total effect value of childhood adversity on sleep quality was 0.208 (95% CI: 0.146, 0.270), with a direct effect value of 0.066 (95% CI: 0.006, 0.130), accounting for 31.73% of the total effect, and a total indirect effect value of 0.142 (95% CI: 0.119, 0.170), accounting for 68.27% of the total effect. (2) The mediating effect of anxiety on the association between childhood adversity and sleep quality was significant, with a path effect value of 0.096 (95% CI: 0.078, 0.119), accounting for 46.15% of the total effect. (3) The mediating effect of negative coping on the association between childhood adversity on sleep quality was significant, with a path effect value of 0.024 (95% CI: 0.014, 0.037), accounting for 11.54% of the total effect. (4) The multiple mediating effects of anxiety and negative coping on the association between childhood adversity on sleep quality were also significant, with a pathway effect value of 0.022 (95% CI: 0.017, 0.028), accounting for 10.58% of the total effect (Table 3).

**Table 3:** Mediation analysis of childhood adversity and sleep quality

Pathway		Effect	SE	95%CI	Proportion(%)
Total effect	ACEs→PSQI	0.208	0.031	0.146, 0.270	100
Direct effect	ACEs→PSQI	0.066	0.032	0.006, 0.130	31.73
Total indirect effects	ACEs→PSQI	0.142	0.013	0.119,0.170	68.27
Indirect effect 1	ACEs→SAS→PSQI	0.096	0.01	0.078, 0.119	46.15
Indirect effect 2	ACEs→Negative coping→PSQI	0.024	0.006	0.014, 0.037	11.54
Indirect effect 3	ACEs→SAS→Negative coping→PSQI	0.022	0.003	0.017, 0.028	10.58

## Discussion

### Current status and influencing factors of sleep quality in older adults

The proportion of older adults with poor sleep quality (PSQI score ≥ 7) was 48.15%, which is similar to the results of previous studies [15, 16]. Due to gradual aging, the sleep-wake cycle of the older adults is disordered, and the efficiency of the circadian rhythm mechanism is reduced, which leads to changes in their sleep duration, sleep architecture, and sleep depth [12]. Furthermore, the occurrence of a variety of sleep problems such as sleep disruption, early sleep onset, and early awakening [47–49], result in a general decline in the sleep quality of older adults. We also found that gender, educational level, marital status, residency status, and chronic diseases were influencing factors of sleep quality. First, women have poorer sleep quality than men, which is in accordance with the established viewpoint [50, 51]. Poor sleep quality and an increased risk of sleep disorders in older women may be due to the following reasons: (1) women are at a disadvantage in terms of socioeconomic factors, such as education and personal income [52]; (2) women are more susceptible to somatic [53] and psychiatric [54, 55] disorders than men; and (3) women experience changes in secreted reproductive hormones [56]. Second, differences in sleep quality among older adults with different educational levels may be due to the fact that well-educated older adults have a higher sense of wellness and are more likely to access healthcare knowledge, which in turn leads to a better sleep state [57]. Third, the poorer sleep quality in widowed older adults and those living alone than in others may be related to loneliness and lack of social support leading to mood disorders, which in turn may cause reduced sleep efficiency and quality [58]. Finally, having a chronic disease is also a risk factor for poor sleep quality in older adults, which may be related to the physical discomfort caused by chronic diseases, the side effects of medications, and the associated financial pressure and psychological burden [59].

### Direct effect of childhood adversity on sleep quality in older adults

The present study found that childhood adversity had a direct effect on sleep quality. Early life experiences, such as abuse, poverty, or the death of a parent, can affect sleep not only in childhood and adolescence but also in adulthood [60, 61]. Childhood is an important phase for significant development of the hypothalamic-pituitary-adrenal (HPA) axis and the brain [58], and adverse events experienced during childhood can lead to long-term changes in the HPA axis response to stress (e.g., hyperactivity) and interfere with normal neurodevelopment in childhood and adolescence [62], increasing the risk of developing psychiatric disorders such as depression and post-traumatic stress disorder, which indirectly affect sleep in adulthood [63]. In addition, people exposed to ACEs are more likely to adopt unhealthy lifestyles and behaviors [64, 65], and these changes may directly affect the sleep-wake cycle and lead to sleep problems.

### Mediating effect of anxiety between childhood adversity and sleep quality in older adults

Sleep problems are not only a precursor but also a consequence of mental illness [66, 67]. Our study found that anxiety could partially explain the relationship between childhood adversity and sleep disorders. Extensive studies have confirmed that exposure to adverse experiences in early life can increase an individual’s risk of developing psychiatric disorders such as anxiety and depression [68, 69]. Anxiety is thus associated with a variety of sleep problems, with higher levels of anxiety corresponding to more severe sleep disorders [25, 70, 71]. Furthermore, anxiety has been found to mediate the effects of childhood adversity on sleep quality. For example, Amarneh found that elevated levels of anxiety sensitivity may explain the relationship between child maltreatment and adult sleep disorders among psychiatric hospitalizations [72]. Haimov found that COVID-19-related anxiety mediated the association between the number of childhood adversities and adult sleep quality [73]. The findings of our study further support the mediating role of anxiety on the effects of childhood adversity on sleep quality in older adults, suggesting that actively intervening in older adults’ anxiety states may mitigate the effects of childhood adversity on their sleep quality.

### Mediating effect of negative coping between childhood adversity and sleep quality in older adults

Our results also identified a significant mediating effect of negative coping in the action of childhood adversity on sleep quality. Individuals’ exposure to environmental stressors early in life can compromise their adaptive coping strategies [74] and thus further affect sleep [75]. This result can be explained by the theory of stress. This theory states that when facing stressful events, people may take measures to disengage from threatening stimuli and generate associated thoughts and emotions (i.e., reducing activity and sleeping longer to minimize exposure to the stressor and the associated maladaptive emotions and thoughts) as well as adopt emotion-focused coping (i.e., regulating emotional responses to problems). However, such approaches may increase alertness and thus produce physiological arousal, disrupting or reducing sleep, which in turn affects sleep quality [76].

Finally, we founded that childhood adversity affected sleep quality in older adults through anxiety and negative coping. As mentioned above, stressful life events in childhood are associated with an increased risk of anxiety disorders in adulthood. Under the influence of such negative emotions, individuals are more inclined to adopt negative coping, which in turn affects the sleep quality in older adults. The above results facilitate a deeper understanding of the relationships among childhood adversity, anxiety, negative coping, and sleep quality and provide clues for exploring the potential mechanisms of how childhood adversity affects sleep quality in older adults.

### Research limitations

In this study, the theoretical structural equation model fit the data well and provided an epidemiologic basis for the associations among childhood adversity, anxiety, negative coping, and sleep quality. However, there are several limitations. First, the results for the main variables in this study were obtained via self-report from the respondents and thus may be subject to unavoidable recall bias. Second, this study utilized a cross-sectional research design, which does not allow for a more precise determination of the causal relationship between variables. Third, this study explored the relationship between ACEs and PSQI scores but did not determine a dose-response relationship or whether different types of childhood adversities have different effects on sleep quality. Finally, the effects of drugs (such as antidepressants and anti-inflammatory drugs) on sleep quality were ignored in this study.

## Conclusions

To sum up, anxiety and negative coping not only had direct effects on sleep quality but also played mediating roles in the association between childhood adversity and sleep quality, with a chained multiple mediating effect. These findings suggest that timely intervention for anxiety symptoms and negative coping states in older adults may mitigate the negative impact of childhood adversity on sleep quality.

## Data Availability

The datasets used and analyzed during the current study are available from the corresponding author on reasonable request.

## Abbreviations

ACEs: Adverse Childhood Experiences
PSQI: the revised Chinese-version Pittsburgh Sleep Quality Index
SAS: Self-Rating Anxiety Scale
TCSQ: Trait Coping Style Questionnaire
SEM: Structural equation modelling
OR: odds ratio
CI: confidence interval
RMSEA: root mean square error of approximation
HPA: the hypo-thalamic pituitary adrenal axis
